# Optimal Cut-Off Points of Fasting Plasma Glucose for Two-Step Strategy in Estimating Prevalence and Screening Undiagnosed Diabetes and Pre-Diabetes in Harbin, China

**DOI:** 10.1371/journal.pone.0119510

**Published:** 2015-03-18

**Authors:** Chundan Bao, Dianfeng Zhang, Bo Sun, Li Lan, Wenxiu Cui, Guohua Xu, Conglan Sui, Yibaina Wang, Yashuang Zhao, Jian Wang, Hongyuan Li

**Affiliations:** 1 Department of Epidemiology, Public Health College, Harbin Medical University, Harbin, Heilongjiang Province, P. R. China; 2 Department of Chronic Noncommunicable Disease Control and Prevention, Harbin Center for Disease Control and Prevention, Harbin, Heilongjiang Province, P. R. China; Università di Milano, ITALY

## Abstract

To identify optimal cut-off points of fasting plasma glucose (FPG) for two-step strategy in screening abnormal glucose metabolism and estimating prevalence in general Chinese population. A population-based cross-sectional study was conducted on 7913 people aged 20 to 74 years in Harbin. Diabetes and pre-diabetes were determined by fasting and 2 hour post-load glucose from the oral glucose tolerance test in all participants. Screening potential of FPG, cost per case identified by two-step strategy, and optimal FPG cut-off points were described. The prevalence of diabetes was 12.7%, of which 65.2% was undiagnosed. Twelve percent or 9.0% of participants were diagnosed with pre-diabetes using 2003 ADA criteria or 1999 WHO criteria, respectively. The optimal FPG cut-off points for two-step strategy were 5.6 mmol/l for previously undiagnosed diabetes (area under the receiver-operating characteristic curve of FPG 0.93; sensitivity 82.0%; cost per case identified by two-step strategy ¥261), 5.3 mmol/l for both diabetes and pre-diabetes or pre-diabetes alone using 2003 ADA criteria (0.89 or 0.85; 72.4% or 62.9%; ¥110 or ¥258), 5.0 mmol/l for pre-diabetes using 1999 WHO criteria (0.78; 66.8%; ¥399), and 4.9 mmol/l for IGT alone (0.74; 62.2%; ¥502). Using the two-step strategy, the underestimates of prevalence reduced to nearly 38% for pre-diabetes or 18.7% for undiagnosed diabetes, respectively. Approximately a quarter of the general population in Harbin was in hyperglycemic condition. Using optimal FPG cut-off points for two-step strategy in Chinese population may be more effective and less costly for reducing the missed diagnosis of hyperglycemic condition.

## Introduction

Pre-diabetes (either impaired fasting glucose [IFG] or impaired glucose tolerance [IGT]) is a relatively high-risk state for diabetes.[1] Pre-diabetes and diabetes carry risk of diabetes complications and cardiovascular disease, especially in people who remain with abnormal glucose metabolism despite intensive intervention.[2–4] In 2003, American Diabetes Association revised the lower cut-point of fasting plasma glucose (FPG), which redefined IFG from 6.1 mmol/l to 5.6 mmol/l (ADA criteria).[4] However, World Health Organization and some other organizations adopted a cut-point of 6.1mmol/l for FPG as the upper limit of normoglycaemia as before (WHO criteria).[5] In China, the prevalences of pre-diabetes using WHO criteria and diabetes reached 15.5% and 9.7% respectively, and 60.7% of the people with diabetes had been undiagnosed.[6] Accordingly, screening for pre-diabetes and undiagnosed diabetes in an early stage should be advocated in China.

Both FPG and 2 hour post-load plasma glucose (2-h PG) after a 75-g oral glucose tolerance test (OGTT) have been used as the gold standard to identify individuals with pre-diabetes and diabetes. Since the OGTT cost more demands on participants’ time, FPG is the most widely used test for detecting diabetes and pre-diabetes in some epidemiological studies and screening. However, approximately half of the participants with undiagnosed diabetes and 70% of the participants with pre-diabetes using WHO criteria had isolated increased 2-h PG in Chinese people.[6] FPG alone (a one-step strategy) [7–9] could lead to lower estimate of prevalence and missed diagnosis. A two-step strategy (including an OGTT was conducted only in subjects with IFG) [10–12] as recommended by WHO could reduce underestimate of diabetes relatively rather than pre-diabetes. It was unclear whether two-step strategy could be used to screen pre-diabetes and/or diabetes in Chinese people with a large proportion of isolated increased 2-h PG.

Harbin is the capital of Heilongjiang Province, which is the most northern province of China. A population-based cross-sectional study was conducted to describe the prevalences of diabetes and pre-diabetes in here, which accurately defined by performing FPG and OGTT in all participants. Meanwhile, we identified screening potential of FPG, cost per case identified by two-step strategy, and the optimal FPG cut-points for two-step strategy in screening undiagnosed diabetes and/or pre-diabetes in general Chinese population. We also assessed the accuracy of FPG alone and two-step strategy in measuring prevalences of undiagnosed diabetes and pre-diabetes using ADA criteria or WHO criteria, respectively.

## Methods

### Ethics Statement

The Institutional Review Board of Public Health College, Harbin Medical University approved the study. Written informed consent was obtained from each participant before survey.

### Study population and sampling

The study was conducted between August 2008 and October 2008 in central urban area of Harbin with a population of 3,345,328. A multistage, stratified probability random sampling method was used to obtain a representative sample of the urban residents aged 20 to 74 years in Harbin. First, 5 city districts (Nangang, Daoli, Daowai, Xiangfang, and Pingfang) were typically selected from total of 8 city districts of Harbin. Those city districts which were not selected (Songbei, Hulan, and A’cheng), with a population of 1,405,970, were far away from the central area of Harbin. Second, 3 street districts were randomly selected from each of the 5 sampled city districts. The street districts were sampled based on stratified sampling according to degree of economic development status (high, middle, and low). In the third stage, 2 communities were randomly sampled from each selected street districts. In the final stage, residents aged 20 to 74 years were randomly selected from the selected communities. The sampling proportion within communities was based on the age and sex structure of the selected city district. Only residents who had lived in their current residence for at least 1 year were eligible to participate.

A total of 9,600 eligible residents were selected and invited to participate in the study; 7,939 of them (2,854 men and 5,085 women) completed the survey. The overall response rate was 82.7%: 64.2% for men and 98.7% for women. Twenty six residents, whose data on FPG or 2-h PG were missing, were excluded from analysis, and 7,913 residents (35.9% men; mean [SD] age 49.3±12.3 years) were included in the prevalence analysis. 7,464 residents (35.8% men; mean [SD] age 48.9±12.4 years) were unknown to have diabetes, and were included in the screening test analysis.

### Data collection and examination

All physicians and staff members who conducted the study were trained in the methodology and principles of the study. Data collection was conducted by the physicians in the community health care centers in the participants’ residential area in the morning. For participants unable to go to health care centers, data collection was conducted at their homes. Physicians administered a standard questionnaire to obtain information on demographic characteristics, personal and family diabetes medical history, etc. Participants were asked whether, other than during pregnancy for women, a doctor had ever told them that they suffered diabetes. Height and weight were measured while subjects were not heavy clothing. The body mass index was calculated as weight (in kilograms) divided by the square of the height (in meters). The waist circumference was measured at a point immediately above the iliac crest on the midaxillary line. The obesity was defined as body mass index ≥25 kg/m^2^, and abdominal obesity was defined as waist circumference ≥85 cm for men and ≥80 cm for women.[13]

All the participants were instructed to maintain their usual diet for 3 days before survey. After 10–12 hours of overnight fasting, venous blood samples were collected in the morning for the measurement of FPG. Then each participant underwent a standard 75-g OGTT, and blood samples were drawn at 120 minutes to measure 2-h PG. Simultaneous detection of FPG and 2-h PG was applied using the hexokinase enzymatic method (Amorsino automatic biochemistry analyzer, Mol 300, China) based on blind method by technicians of Center for Disease Control and Prevention.

### Definitions

Diagnosed diabetes was identified by a positive response from the participants to the question in the interview, “Have you ever been told that you suffered diabetes by a doctor?” Participants without diagnosed diabetes until this study were classify as undiagnosed diabetes (FPG ≥7.0 mmol/l and/or 2-h PG ≥11.1 mmol/l), pre-diabetes using ADA criteria (IFG [FPG 5.6 to <7.0 mmol/l] and/or IGT [2-h PG 7.8 to <11.1 mmol/l]), pre-diabetes using WHO criteria (IFG [FPG 6.1 to <7.0 mmol/l] and/or IGT [2-h PG 7.8 to <11.1 mmol/l]), and normal, respectively.[4–5] These were as golden standards in screening test analysis. Undiagnosed diabetes was stratified into three subcategories: (a) isolated fasting diabetes (FPG ≥7.0 mmol/l and 2-h PG <11.1 mmol/l); (b) isolated 2h post-load diabetes (FPG <7.0 mmol/l and 2-h PG ≥11.1 mmol/l); (c) combined fasting and post-load diabetes (FPG ≥7.0 mmol/l and 2-h PG ≥11.1 mmol/l). Pre-diabetes using ADA criteria was also stratified into three subcategories: (a) isolated IFG (FPG 5.6 to <7.0 mmol/l and 2-h PG <7.8 mmol/l); (b) isolated IGT (FPG <5.6 mmol/l and 2-h PG 7.8 to <11.1 mmol/l); (c) combined IFG and IGT (FPG 5.6 to <7.0 mmol/l and 2-h PG 7.8 to <11.1 mmol/l). The same applied to the classification of pre-diabetes using WHO criteria. Two-step strategy was that the individuals with an increased FPG (≥ the FPG cut-points and < the value used to define IFG or undiagnosed diabetes) [14,15] were given an OGTT after all subjects first completed FPG test.

### Statistical analysis

The prevalences of diabetes and pre-diabetes were calculated for the overall study subjects and for subgroups according to age and sex. Weights that adjusted for different sampling probabilities and the deviations in sex and age between the sample and the total study subjects were routinely used in all analyses to represent the total population aged 20 to 74 years on the basis of the study sampling scheme and Harbin’s urban area population data in 2008. Standard errors and confidence intervals (CI) were estimated with the Taylor series linearization.[16] Standardized prevalences were calculated by the direct method using China adult population aged 20 to 74 from the 2005 National Sample Survey of 1% of Population as the standard population. Prevalence analysis was performed with SURVEYFREQ Procedure that was appropriate to the complex multistage survey design in SAS 9.1.3 software (SAS Institute Inc., Shanghai, China).

We used SPSS version 13.0 for screening test analysis. A *P* value less than 0.05 was considered statistically significant. We used the method described by Hanley and McNeil to compare the area under the receiver-operating characteristic curves (AUC) for FPG and 2-h PG. Screening potential of FPG was described by sensitivity, specificity, likelihood ratio, post-test probability, etc. Post-test probability was calculated from pre-test probability and likelihood ratio. Pre-test probability of an individual with known characteristics was estimated from the prevalence of the abnormal glucose metabolism in known characteristics. The point with maximization of the sum of sensitivity and specificity was selected as optimal cut-off point. The total cost of two-step strategy was estimate by medical and non-medical cost. The medical, non-medical and total cost for one time FPG (OGTT) test were 7.8, 8.3, 16.1 (11.8, 27.5, 39.3) ¥, respectively.[14] The cost-effectiveness were calculated as follows

Total cost = (all subjects ×one time FPG cost + subjects with increased FPG ×one time OGTT cost)

The cost-effectiveness (cost per case identified) of two-step strategy = total cost ÷ undiagnosed diabetes and/or pre-diabetes cases identified.

## Results

### Prevalence of diabetes

The prevalences of undiagnosed diabetes, diagnosed diabetes, and total diabetes were 8.3%, 4.4%, and 12.7%, respectively. The prevalences of isolated fasting diabetes and diagnosed diabetes were similar between men and women; the prevalences of isolated 2h post-load diabetes, combined fasting and post-load diabetes, undiagnosed diabetes, and total diabetes were slightly higher in men than those in women, but the differences were not statistically significant except for combined fasting and post-load diabetes (*P* = 0.0427). However, the prevalences of these kinds of diabetes at age 40–59 years were significantly higher in men than those in women. In contrast, the sex differences of prevalences of these kinds of diabetes in all the other age groups were not statistically significant.(S1 Table)

The prevalences of isolated 2h post-load diabetes, combined fasting and post-load diabetes, undiagnosed diabetes, diagnosed diabetes, and total diabetes increased with age and peaked at age 60–74 years in men and women (*P* <0.0001). However, the prevalence of isolated fasting diabetes was fluctuated with age. (S1 Table)

### Awareness, treatment and control of diabetes

Of those participants with diabetes, 34.8% were aware of their diabetes, 31.5% (90.7% of participants who were aware of diabetes) were taking medication or nonpharmacological interventions, and 10.8% (31.1% of those treated) were controlled (FPG <7.0 mmol/l, and 2-h PG <11.1 mmol/l). The proportions of awareness, treatment and control of diabetes were similar between men and women.(data not shown) The proportion of undiagnosed diabetes in total diabetes was 65.2%. The proportion of undiagnosed diabetes was significantly higher in men than that in women (68.4% vs. 61.4%, respectively; *P* = 0.0213) and decreased with age (*P* = 0.0080). The proportion of undiagnosed diabetes in men at age 20–39 years was significantly higher than those at other age groups and in women.(S1 Table)

### Prevalence of pre-diabetes

Twelve percent (6.3% with IFG and 7.8% with IGT, 12.9% for men and 11.2% for women) or 9.0% (2.2% with IFG and 7.8% with IGT, 9.3% for men and 8.8% for women) of participants were diagnosed with pre-diabetes using ADA criteria or WHO criteria, respectively. The prevalences of isolated IFG, isolated IGT, combined IFG and IGT, total pre-diabetes, and total diabetes and pre-diabetes using ADA criteria or using WHO criteria were slightly higher in men than those in women but without statistics significant except for combined IFG and IGT using WHO criteria. The prevalences of them using ADA criteria were significantly higher in men than those in women at age 40–59 years. Meanwhile, the prevalences of isolated IGT, total pre-diabetes, and total diabetes and pre-diabetes using WHO criteria were also higher in men than those in women at age 40–59 years (*P* <0.05). The prevalences of isolated IGT, pre-diabetes, and total diabetes and pre-diabetes increased with age and peaked at age 60–74 years using ADA criteria and WHO criteria (*P* <0.0001). However, isolated IFG and Combined IFG and IGT were fluctuated with age.(S2 Table)

### Standardized prevalences

The standardized prevalences of diabetes, pre-diabetes, and total diabetes and pre-diabetes were 12.4%, 11.5%, and 23.9% using ADA criteria and 12.4%, 8.6%, and 21.0% using WHO criteria based on the 2005 National Sample Survey of 1% of Population.

### The screening test analysis

**Fig 1:**
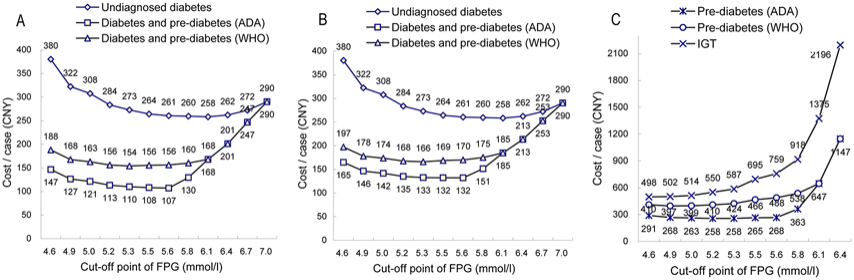
The total cost per case identified by two-step strategy at different FPG cut-points for further OGTT test. In Fig.1A, further OGTT was not conducted for subjects with FPG ≥5.6 mmol/l (≥6.1mmol/l) in the case of screening for both diabetes and pre-diabetes using ADA criteria (WHO criteria). In Fig.1B and 1C, further OGTT was not conducted for subjects with FPG ≥7.0 mmol/l in the case of screening for undiagnosed diabetes or both diabetes and pre-diabetes or pre-diabetes alone or IGT.

The optimal FPG cut-off points were 5.6 mmol/l for previously undiagnosed diabetes, 5.3 mmol/l for both diabetes and pre-diabetes or pre-diabetes using ADA criteria, 5.0 mmol/l for pre-diabetes using WHO criteria, and 4.9 mmol/l for IGT. The AUCs and sensitivities of these points were lower for FPG than for 2-h PG in screening both diabetes and pre-diabetes, undiagnosed diabetes, and pre-diabetes.(Table 1 and S3 Table) Nevertheless, the AUCs for FPG (sensitivity, Specificity) were greater than 0.7 (60%, 70%), and Kappa values between optimal FPG cut-off points and gold standards were statistically significant (*P* <0.001). Therefore further OGTT (two-step strategy) should be conducted to increase specificity for screening diabetes and/or pre-diabetes. Using the optimal FPG cut-off points for screening pre-diabetes using WHO criteria or IGT alone, Kappa values (<0.4) and specificity (<80%) were lower, and OGTT alone (one-step strategy) should be better conducted.(Table 1) The total costs per case of these points were relatively lower.(Fig. 1) 5.6 mmol/l for previously undiagnosed diabetes and 5.3 mmol/l for both diabetes and pre-diabetes using ADA criteria were with the least medical cost per case. Medical and total cost per case of these points were both diabetes and pre-diabetes (ADA criteria), ¥51, ¥110, both diabetes and pre-diabetes (WHO criteria), ¥69, ¥154, pre-diabetes alone (ADA criteria), ¥112, ¥258, diabetes alone, ¥117, ¥261, pre-diabetes alone (WHO criteria), ¥166, ¥399, IGT alone, ¥205, ¥502, ascendingly.(S4 Table) The optimal FPG cut-off points for previously undiagnosed diabetes were 5.3 mmol/l (sensitivity 86.1%; specificity 81.5%) at age 60–74 years and 5.6 mmol/l at age<60 years (sensitivity 84.6%; specificity 92.1%), respectively. The optimal FPG cut-off points for IGT were 4.9 mmol/l in men (sensitivity 60.5%, specificity 73.2%) and women (sensitivity 62.2%, specificity 75.2%).

**Table 1:** AUCs and screening potential for FPG alone in screening undiagnosed diabetes and/or pre-diabetes with optimal cut-off points.

Optimal cut-off point (mmol/l)	Positive/Negative*	Sensitivity (%)	Specificity (%)	Likelihood ratio (%)		Predictive value (%)		Kappa	AUC (95% CI)	Post-test probability (%)			
				Positive	Negative	Positive	Negative			Men	Age >40 years	Obesity	Abdominal obesity
> = 5.6 for undiagnosed diabetes	696/6768	82.0	91.6	9.8	0.2	50.2	98.0	0.57	0.93(0.92–0.94)^§^	50.4	55.0	56.3	57.2
> = 5.3 for diabetes and pre-diabetes^†^ (ADA criteria)	1728/5736	72.4	94.4	12.8	0.3	79.4	91.9	0.69	0.89(0.88–0.90)^§^	78.6	82.5	83.3	83.2
> = 5.3 for diabetes and pre-diabetes^‡^ (WHO criteria)	1478/5986	68.0	90.2	6.9	0.4	63.0	91.9	0.57	0.86(0.84–0.87)^§^	61.3	67.5	68.9	68.2
> = 5.3 for pre-diabetes^†^ (ADA criteria)	1032/5736	62.9	94.1	10.6	0.4	65.7	93.4	0.58	0.85(0.83–0.87)^§^	61.1	66.5	67.5	66.7
> = 5.0 for pre-diabetes^‡^ (WHO criteria)	782/5986	66.8	75.8	2.8	0.4	26.5	94.6	0.28	0.78(0.76–0.80)^§^	22.3	27.6	28.9	27.1
> = 4.9 for IGT	674/6094	62.2	73.8	2.4	0.5	20.8	94.6	0.19	0.74(0.73–0.75)	17.3	22.0	23.2	21.2

AUCs, the area under the receiver-operating characteristic curves; FPG, fasting plasma glucose.

*Number of participants based on golden standard. Pre-diabetes includes IFG and/or IGT.

^†^IFG using ADA criteria, FPG 5.6 to <7.0 mmol/l;

^‡^IFG using WHO criteria, FPG 6.1 to <7.0 mmol/l; IGT, 2-h PG 7.8 to <11.1 mmol/l.

^§^ *P* <0.001 compared with area under curve of 2 hour post-load plasma glucose.

The pre-test probabilities of undiagnosed diabetes (pre-diabetes using ADA criteria, pre-diabetes using WHO criteria) were 11.6% (16.4%, 12.7%) for obesity and 12.0% (15.9%, 11.7%) for abdominal obesity. The post-test probabilities of them were shown in Table 1. Positive likelihood ratios of different FPG cut-off points were shown in Table 2.

**Table 2:** Positive likelihood ratios of different FPG cut-off points.

FPG (mmol/l)	Number of participants based on 2-h PG (mmol/l)				Positive likelihood ratios				
	Total	<7.8	> = 7.8 and <11.1	> = 11.1	Diabetes	Diabetes and pre-diabetes* (ADA criteria)	Diabetes and pre-diabetes^†^ (WHO criteria)	Pre-diabetes* (ADA criteria)	Pre-diabetes^†^ (WHO criteria)
<4.5	3237	3072	138	27	0.1	0.2	0.2	0.2	0.3
4.5–4.8	1637	1480	126	31	0.2	0.4	0.4	0.5	0.7
4.9–5.1	824	702	95	27	0.3	0.6	0.7	0.8	1.0
5.2–5.4	517	390	95	32	0.6	1.1	1.3	1.4	1.9
5.5	128	92	25	11	0.9	1.3	1.6	1.5	2.1
5.6–6.0	406	250	99	57	1.6	-	2.5	-	3.0
6.1–6.9	301	108	96	97	4.6	-	-	-	-
7.0-	414	57	40	317	-	-	-	-	-
Total	7464	6151	714	599	-	-	-	-	-

FPG, fasting plasma glucose; 2-h PG, 2 hour post-load plasma glucose; Pre-diabetes includes IFG and/or IGT.

*IFG using ADA criteria, FPG 5.6 to <7.0 mmol/l;

^†^IFG using WHO criteria, FPG 6.1 to <7.0 mmol/l; IGT, 2-h PG 7.8 to <11.1 mmol/l.

### The accuracy of FPG alone in measuring prevalence of undiagnosed diabetes and pre-diabetes

Among those classified as normal by FPG, 91.2% or 89.6% were classified equally by 2-h PG using ADA criteria or WHO criteria, respectively. Of those people with IFG using WHO criteria, 32.4% had diabetes, 31.0% had combined IFG and IGT, and 36.6% had isolated IFG.(Table 3)

**Table 3:** Classification of FPG alone and 2-h PG alone for abnormal glucose metabolism in urban Harbin of China, 2008 (%, 95% CI).

Diagnosis based on FPG	Diagnosis based on 2-h PG	Distribution across 2-h PG categories by FPG diagnostic category		Distribution across FPG and 2-h PG categories	
		ADA criteria*	WHO criteria^†^	ADA criteria*	WHO criteria^†^
Undiagnosed diabetes	Undiagnosed diabetes	78.3(72.1–84.5)	78.3(72.1–84.5)	3.9(2.7–5.1)	3.9(2.7–5.1)
	IGT	8.5(4.2–12.8)	8.5(4.2–12.8)	0.4(0.2–0.6)	0.4(0.2–0.6)
	Normal	13.2(10.0–16.4)	13.2(10.0–16.4)	0.7(0.5–0.9)	0.7(0.5–0.9)
IFG	Undiagnosed diabetes	21.8(15.5–28.0)	32.4(22.3–42.4)	1.7(1.1–2.4)	1.1(0.7–1.4)
	IGT	26.6(21.5–31.6)	31.0(20.4–41.5)	2.1(1.4–2.9)	1.0(0.4–1.6)
	Normal	51.6(44.0–59.4)	36.6(28.9–44.3)	4.2(2.7–5.7)	1.2(0.7–1.7)
Normal	Undiagnosed diabetes	1.9(1.5–2.3)	2.6(2.0–3.2)	1.6(1.2–1.9)	2.2(1.7–2.8)
	IGT	6.9(5.3–8.5)	7.8(6.2–9.4)	5.7(4.5–6.8)	6.8(5.6–8.1)
	Normal	91.2(89.5–92.9)	89.6(87.6–91.6)	75.2(70.2–80.2)	78.3(73.9–82.5)
Diagnosed diabetes		-	-	4.4(2.8–6.0)	4.4(2.8–6.0)
Total		-	-	100.0	100.0

FPG, fasting plasma glucose; 2-h PG, 2 hour post-load plasma glucose; diagnosed diabetes, determined by self-report on interview.

*IFG using ADA criteria, FPG 5.6 to <7.0 mmol/l;

^†^IFG using WHO criteria, FPG 6.1 to <7.0 mmol/l; IGT, 2-h PG 7.8 to <11.1 mmol/l.

The propotions of isolated 2h post-load diabetes in undiagnosed diabetes (isolated IGT in total pre-diabetes) were relatively higher in all the age groups and in men and women, especially at age 60–74 years (using WHO criteria).(S1 and S2 Tables)

## Discussion

Two national prevalence surveys of diabetes suggested that there were marked geographical differences in the prevalence of diabetes in China, with much higher prevalence in urban areas and in northern China.[6,7] Accordingly, measuring prevalence and screening diabetes should be advocated more in here. The study was a large-scale population-based survey. All participants underwent an OGTT. The diabetes and pre-diabetes were defined based on performing FPG and 2-h PG from an OGTT. This was designed to provide more strong evidence that assessed the performance of screening strategy in general population rather than for high risk population and more accurate estimates of the prevalences of diabetes and pre-diabetes than previous studies in Northern China.[8,17] Furthermore, the standardized training and the quality-control procedures were completed to ensure the validity of the results during the study.

The prevalence of diabetes in Harbin was 12.7%, which was similar to that in U.S. in 2005 when using the U.S. 2000 Census population as standard population (Harbin 12.2% vs. U.S. 12.6%) and higher than the mean prevalence in China in 2008 (standardized prevalence based on China population in 2005: 12.4% vs. 9.7%).[6,18] The proportion of undiagnosed diabetes was 65.2%, higher than that in U.S. in 2005 (39.8%) [18] and China in 2008 (60.7%).[6] In addition, the prevalence of pre-diabetes in Harbin was lower than that in U.S. in 2005 (standardized prevalence based on the U.S. 2000 Census population using ADA criteria: Harbin 11.9% vs. U.S. 29.0%) or China in 2008 (standardized prevalence based on China population in 2005 using WHO criteria: Harbin 8.6% vs. China 15.5%).[6,18]

Although the awareness, treatment and control of diabetes were relatively low in total diabetes patients, 90.7% of diagnosed diabetes were treated; among those treated, 31.1% were controlled. Therefore, screening diabetes and pre-diabetes, and improving the awareness would be more urgently needed for the intervention of hyperglycemic condition than other strategies in the adult population in Northern China.

The accuracy (sensitivity, AUC of FPG) and cost-effectiveness of the optimal FPG cut-points for two-step strategy were relatively better. Especially in screening diabetes, the accuracy of 5.6 mmol/l as cut-point was better than other tests (A1C, fasting capillary glucose, and Chinese diabetes risk score).[19–21] In screening pre-diabetes using WHO criteria in Chinese people, the accuracy of 5.0 mmol/l as cut-point was better than A1C and fasting capillary glucose.[19] Our study demonstrated higher optimal FPG cut-off points in screening undiagnosed diabetes than that in the paper by Ye et al [14] (5.6 mmol/l vs. 5.4 mmol/l). Meanwhile, our study suggested a lower cost using optimal FPG cut-off points in screening undiagnosed diabetes than that in the paper by Ye et al [14] (¥261 vs. ¥615), because of higher prevalence of diabetes (12.7% vs. 8.8%) and the proportion of undiagnosed diabetes in total diabetes (65.2% vs. 40.9%) probably. According to the goal of screening (diabetes alone or both diabetes and pre-diabetes or pre-diabetes alone or IGT alone), criteria (2003 ADA criteria or 1999 WHO criteria) and funds (medical cost or total cost), different FPG cut-points should be chosen.

In China, more and more people receive health examination paid by the goverment or medical insurance system or individual, and FPG is a routine examination in health examination center or community health care center. The optimal FPG cut-off points for census of diabetes and/or pre-diabetes in general population would guide physician decision-making in health examination center or community health care center. Meanwhile positive likelihood ratio and post-test probability allow the physician to better interpret the results of FPG and predict the likelihood of a true positive result.

If diabetes or pre-diabetes were identified by FPG alone as a one-step strategy in our survey, the prevalence exhibited underestimates of 26.0% for total diabetes, 40.0% for undiagnosed diabetes, 47.2% for pre-diabetes using ADA criteria, and 75.4% for pre-diabetes using WHO criteria. If further OGTT was conducted for subjects with FPG 5.6 to <7.0 mmol/l (6.1 to <7.0 mmol/l) as a two-step strategy, the prevalence exhibited underestimates of 12.2% (17.3%) for total diabetes and 18.7% (26.5%) for undiagnosed diabetes.(S1 and S2 Table) The similar findings were found by the DECODA, which reported that using the two-step strategy would fail to detect every fourth individual with diabetes and every second individual with IGT in Asia.[22] Using the two-step strategy for pre-diabetes using WHO criteria (FPG 5.3 mmol/l as cut-point) or ADA criteria (FPG 5.0 mmol/l as cut-point), the underestimates of prevalence reduced to nearly 38%. (S2 Table)

Previous studies have reported a 2–3-fold increase in prevalence of IFG using the new ADA recommended criteria compared with WHO criteria.[23,24] In our survey, the prevalence of IFG increased from 2.2% (WHO criteria) to 6.3% (ADA criteria). The risk of diabetes and coronary heart disease for IGT was higher than that for IFG,[22,23] and IGT carried more risk of death than IFG.[25] In our study, people with IGT had lower fasting plasma glucose level than those with IFG in men and women using ADA criteria or using WHO criteria.(S5 Table) Therefore, it can be inferred that the coronary heart disease risk for lower normal fasting plasma glucose level is higher than that for IFG, which is in agreement with other study.[26] ADA reduced the lower FPG cut-point, in part to ensure that prevalence of IFG was similar to that of IGT.[4] However, in our survey, underestimate of isolated IGT decreased only from 6.8% (WHO criteria) to 5.7% (ADA criteria), and lower FPG cut-off point (4.9 mmol/l) for two-step strategy should be used to improve sensitivity. There was also some evidence that diabetes diagnosed solely on the basis of 2–h PG was associated with a worse prognosis than diabetes diagnosed in the sight of FPG alone for mortality and retinopathy.[27,28] Therefore, a two-step strategy or an OGTT alone should be necessary for screening diabetes and pre-diabetes in the Chinese population, because it could detect isolated 2h post-load diabetes and isolated IGT, which were found in large portions in our study and the other study.[6] Although the sensitivity and cost-effectiveness of two-step strategy for screening IGT (4.9 mmol/l as FPG cut-off points) was relatively lower, the optimal cut-off point in screening IGT could provide the basis for the screening of coronary heart disease risk factors. The study [29] suggested IGT independently predicted coronary heart disease risk in women. Therefore, optimal FPG cut-off points for two-step strategy in women in screening IGT should be identified, but the sex difference in the optimal cut-off point in screening IGT have not been found.

The study suffered from some limitations. There was a lower response rate among men than among women in this survey. The proportion of those that had never measured blood glucose among participants was 65.0%, and there was no difference between men and women (63.8% vs. 66.3%); the distribution of “How long ago have you been measured blood glucose recently?” (less than 30 days, 1–6 months, 7–12 months, and more than one year) also was similar between men and women (data not shown); the proportion of undiagnosed diabetes in men was higher than that in women. These suggested that the non-response among men was not caused by awareness of their diabetes, and in some extent the low response rate in men may not affect the prevalence of study subjects. Consequently the possibility of selection bias was minimised.

In conclusion, approximately a quarter of the general adult population has hyperglycemic condition, and diabetes has become a major public health challenge in Harbin, Northern China. The prevalences of diabetes and pre-diabetes at age 40–59 years were higher in men than those in women, and special attention should be paid to men aged 40 to 59 years. No matter which criteria should be used (ADA criteria or WHO criteria), greater efforts to use optimal FPG cut-off points for two-step strategy and identify those with IGT and with isolated 2h post-load diabetes in general Chinese population may be more effective and less costly for reducing the missed diagnosis of hyperglycemic condition.

## Supporting Information

S1 Table Prevalence of diabetes, proportion of undiagnosed diabetes in total diabetes, proportion of isolated 2h post-load diabetes in undiagnosed diabetes, and missed diagnosis of undiagnosed diabetes using optimal FPG cut-off point for two-step strategy by age and sex, in urban Harbin of China, 2008 (%, 95% CI). (DOC)

S2 Table Prevalence of pre-diabetes, proportion of isolated IGT in total pre-diabetes, and missed diagnosis of pre-diabetes using optimal FPG cut-off points for two-step strategy by age and sex based on ADA and WHO criteria, in urban Harbin of China, 2008 (%, 95% CI). (DOC)

S3 Table AUCs and screening potential for 2-h PG alone in screening undiagnosed diabetes and/or pre-diabetes with optimal cut-off points. (DOC)

S4 Table The cost-effectiveness of two-step strategy at different fasting plasma glucose cut-off points in screening IGT, undiagnosed diabetes and/or pre-diabetes. (DOC)

S5 Table Mean FPG and mean 2 hour post-load plasma glucose in OGTT by group of plasma glucose categories and sex based on ADA and WHO criteria. (mmol/l, 95% CI). (DOC)

## References

[1] de Vegt F, Dekker JM, Jager A, Hienkens E, Kostense PJ, Stehouwer CD, et al Relation of impaired fasting and postload glucose with incident type 2 diabetes in a Dutch population: The Hoorn Study. Jama 2001;285: 2109–2113. 11311100 10.1001/jama.285.16.2109

[2] Ford ES, Zhao G, Li C. Pre-diabetes and the risk for cardiovascular disease: a systematic review of the evidence. J Am Coll Cardiol 2010;55: 1310–1317. 10.1016/j.jacc.2009.10.060 20338491

[3] Diabetes Prevention Program Research Group. The prevalence of retinopathy in impaired glucose tolerance and recent-onset diabetes in the Diabetes Prevention Program. Diabet Med 2007;24: 137–144. 17257275 10.1111/j.1464-5491.2007.02043.x PMC2267935

[4] American Diabetes Association. Diagnosis and classification of diabetes mellitus. Diabetes Care 2011;34 Suppl 1: S62–69. 10.2337/dc11-S062 21193628 PMC3006051

[5] World Health Organization, International Diabetes Federation. Definition and diagnosis of diabetes mellitus and intermediate hyperglycaemia: report of a WHO/IDF consultation. Geneva: World Health Organization, 2006 Available: http://www.who.int/diabetes/publications/Definition%20and%20diagnosis%20of%20diabetes_new.pdf. Accessed 2008 Jul 21.

[6] Yang W, Lu J, Weng J, Jia W, Ji L, Xiao J, et al Prevalence of diabetes among men and women in China. N Engl J Med 2010;362: 1090–1101. 10.1056/NEJMoa0908292 20335585

[7] Gu D, Reynolds K, Duan X, Xin X, Chen J, Wu X, et al Prevalence of diabetes and impaired fasting glucose in the Chinese adult population: International Collaborative Study of Cardiovascular Disease in Asia (InterASIA). Diabetologia 2003;46: 1190–1198. 12879248 10.1007/s00125-003-1167-8

[8] Zhao JB, Zhao YJ, Fu SY, Wang FM, Yang LT. A cross-sectional study on impaired fasting glycaemia and diabetes mellitus in residents from Nangang district, Harbin city. Zhonghua Liu Xing Bing Xue Za Zhi 2009;30: 110–114. 19565866

[9] Kim SM, Lee JS, Lee J, Na JK, Han JH, Yoon DK, et al Prevalence of diabetes and impaired fasting glucose in Korea: Korean National Health and Nutrition Survey 2001. Diabetes Care 2006;29: 226–231. 16443864 10.2337/diacare.29.02.06.dc05-0481

[10] Wang H, Qiu Q, Tan LL, Liu T, Deng XQ, Chen YM, et al Prevalence and determinants of diabetes and impaired fasting glucose among urban community-dwelling adults in Guangzhou, China. Diabetes Metab 2009;35: 378–384. 10.1016/j.diabet.2009.03.006 19665414

[11] Moadab MH, Kelishadi R, Hashemipour M, Amini M, Poursafa P. The prevalence of impaired fasting glucose and type 2 diabetes in a population-based sample of overweight/obese children in the Middle East. Pediatr Diabetes 2010;11: 101–106. 10.1111/j.1399-5448.2009.00534.x 19765232

[12] Melidonis AM, Tournis SM, Kompoti MK, Lentzas IL, Roussou VR, Iraklianou SL, et al Increased prevalence of diabetes mellitus in a rural Greek population. Rural Remote Health 2006;6: 534 16579675

[13] Zhang S, Tong W, Xu T, Wu B, Zhang Y. Diabetes and impaired fasting glucose in Mongolian population, Inner Mongolia, China. Diabetes Res Clin Pract 2009;86: 124–129. 10.1016/j.diabres.2009.07.013 19712989

[14] Ye Z, Cong L, Ding G, Yu M, Zhang X, Hu R, et al Optimal cut-off points for two-step strategy in screening of undiagnosed diabetes: a population-based study in China. PLoS One 2014;9: e87690 10.1371/journal.pone.0087690 24609110 PMC3946449

[15] Zhang P, Engelgau MM, Valdez R, Cadwell B, Benjamin SM, Narayan KM. Efficient cutoff points for three screening tests for detecting undiagnosed diabetes and pre-diabetes: an economic analysis. Diabetes Care. 2005;28: 1321–1325. 15920046 10.2337/diacare.28.6.1321

[16] SAS Institute Inc. SAS/STAT 9.22 User’s Guide. Cary, NC: SAS Institute Inc, 2010.

[17] Wei W, Liu SY, Zeng FF, Yao SP, Zhang HT, Wan G, et al Type 2 diabetes and impaired glucose tolerance in North-China-based rural community adults. Public Health 2010;124: 593–601. 10.1016/j.puhe.2010.05.014 20846702

[18] Cowie CC, Rust KF, Ford ES, Eberhardt MS, Byrd-Holt DD, Li C, et al Full accounting of diabetes and pre-diabetes in the U.S. population in 1988–1994 and 2005–2006. Diabetes Care 2009;32: 287–294. 10.2337/dc08-1296 19017771 PMC2628695

[19] Zhou X, Pang Z, Gao W, Wang S, Zhang L, Ning F, et al Performance of an A1C and fasting capillary blood glucose test for screening newly diagnosed diabetes and pre-diabetes defined by an oral glucose tolerance test in Qingdao, China. Diabetes Care 2010;33: 545–550. 10.2337/dc09-1410 20007941 PMC2827505

[20] Zhang Y, Sun J, Pang Z, Gao W, Sintonen H, Kapur A, et al Evaluation of two screening methods for undiagnosed diabetes in China: an cost-effectiveness study. Prim Care Diabetes. 2013;7: 275–282. 10.1016/j.pcd.2013.08.003 24021478

[21] Zhou X, Qiao Q, Ji L, Ning F, Yang W, Weng J, et al Nonlaboratory-based risk assessment algorithm for undiagnosed type 2 diabetes developed on a nation-wide diabetes survey. Diabetes Care 2013;36: 3944–3952. 10.2337/dc13-0593 24144651 PMC3836161

[22] DECODA Study Group, International Diabetes Epidemiology Group. Cardiovascular risk profile assessment in glucose-intolerant Asian individuals—an evaluation of the World Health Organization two-step strategy: the DECODA Study (Diabetes Epidemiology: Collaborative Analysis of Diagnostic Criteria in Asia). Diabet Med 2002;19: 549–557. 12099957 10.1046/j.1464-5491.2002.00735.x

[23] Tai ES, Goh SY, Lee JJ, Wong MS, Heng D, Hughes K, et al Lowering the criterion for impaired fasting glucose: impact on disease prevalence and associated risk of diabetes and ischemic heart disease. Diabetes Care 2004;27: 1728–1734. 15220254 10.2337/diacare.27.7.1728

[24] Borch-Johnsen K, Colagiuri S, Balkau B, Glumer C, Carstensen B, Ramachandran A, et al Creating a pandemic of prediabetes: the proposed new diagnostic criteria for impaired fasting glycaemia. Diabetologia 2004;47: 1396–1402. 15278279 10.1007/s00125-004-1468-6

[25] The DECODE study group on behalf of the European Diabetes Epidemiology Group. Glucose tolerance and mortality: comparison of WHO and American Diabetes Association diagnostic criteria. Lancet 1999;354: 617–621. 10466661

[26] Onat A, Can G, Cicek G, Dogan Y, Yuksel H. Coronary disease risk and fasting glucose levels in a non-diabetic population. Diabetes Res Clin Pract 2011;91: 220–225. 10.1016/j.diabres.2010.11.035 21208678

[27] Ito C, Maeda R, Ishida S, Harada H, Inoue N, Sasaki H. Importance of OGTT for diagnosing diabetes mellitus based on prevalence and incidence of retinopathy. Diabetes Res Clin Pract 2000;49: 181–186. 10963830 10.1016/s0168-8227(00)00156-x

[28] DECODE Study Group, European Diabetes Epidemiology Group. Is the current definition for diabetes relevant to mortality risk from all causes and cardiovascular and noncardiovascular diseases? Diabetes Care 2003;26: 688–696. 12610023 10.2337/diacare.26.3.688

[29] Onat A, Can G, Cicek G, Ayhan E, Dogan Y, Kaya H. Fasting, non-fasting glucose and HDL dysfunction in risk of pre-diabetes, diabetes, and coronary disease in non-diabetic adults. Acta Diabetol 2013;50: 519–528. 10.1007/s00592-011-0313-x 21769500

