# Supplementary material for: Optimal Cut-Off Points of Fasting Plasma Glucose for Two-Step Strategy in Estimating Prevalence and Screening Undiagnosed Diabetes and Pre-Diabetes in Harbin, China
Source: PLoS One. 2015 Mar 18;10(3):e0119510. doi: 10.1371/journal.pone.0119510 (PMC4364753; doi:10.1371/journal.pone.0119510)
Supplement: S1 Table — (DOC) [file pone.0119510.s001.doc]

**S1 Table. Prevalence of diabetes, proportion of undiagnosed diabetes in total diabetes, proportion of isolated 2h post-load diabetes in undiagnosed diabetes, and missed diagnosis of undiagnosed diabetes using optimal FPG cut-off point for two-step strategy by age and sex, in urban Harbin of China, 2008 (%, 95% CI)**

|  | Undiagnosed diabetes | | | | Diagnosed diabetes | Total diabetes^*^ | Proportion of undiagnosed diabetes in total diabetes | Proportion of isolated 2h post-load diabetes in undiagnosed diabetes | Proportion of isolated 2h post-load diabetes which FPG <5.6 mmol/l in undiagnosed diabetes |
| --- | --- | --- | --- | --- | --- | --- | --- | --- | --- |
|  | Isolated fasting diabetes | Isolated 2h post-load diabetes | Combined fasting and post-load diabetes | Total |  |  |  |  |  |
| Number | 97 | 282 | 317 | 696 | 449 | 1145 | - | - | - |
| Overall |  |  |  |  |  |  |  |  |  |
| 20-74 (n=7913) | 1.1(0.7-1.4) | 3.3(2.6-4.1) | 3.9(2.7-5.1) | 8.3(6.4-10.2) | 4.4(2.8-6.0) | 12.7(10.1-15.3) | 65.2(56.1-74.4) | 40.0(34.6-45.4) | 18.7(13.1-24.3) |
| Sex |  |  |  |  |  |  |  |  |  |
| Men (n=2841) | 1.0(0.5-1.5) | 3.7(2.4-5.1) | 4.7(2.7-6.8)^‡^ | 9.4(5.9-13.0) | 4.4(2.0-6.8) | 13.8(8.4-19.3) | 68.4(59.6-77.1)^‡^ | 39.2(34.0-44.4) | 19.7(13.4-26.1) |
| Women(n=5072) | 1.2(0.7-1.6) | 2.9(2.3-3.6) | 3.1(2.2-3.9) | 7.2(5.8-8.5) | 4.5(2.9-6.1) | 11.7(10.0-13.3) | 61.4(50.5-72.3) | 41.1(33.3-48.9) | 17.2(10.3-24.1) |
| Age-specific groups (years) |  |  |  |  |  |  |  |  |  |
| 20-39 (n=1789) | 0.7(0.0-1.4)^†^ | 1.6(0.5-2.8)^†^ | 2.3(0.5-4.1)^†^ | 4.6(2.3-7.0) | 1.2(0.6-1.8) | 5.9(3.4-8.3) | 79.2(67.5-90.8) | 35.2(11.6-58.7)^†^ | 20.7(6.1-35.3)^†^ |
| 40-59 (n=4573) | 1.4(1.2-1.6) | 3.6(2.7-4.4) | 4.7(3.4-6.0) | 9.6(7.7-11.6) | 5.9(3.4-8.3) | 15.5(12.9-18.2) | 62.1(50.1-74.0) | 36.9(31.8-41.9) | 14.0(9.9-18.0) |
| 60-74 (n=1551) | 1.3(0.7-1.9) | 7.4(6.0-8.8) | 6.3(4.7-7.8) | 15.0(13.0-17.0) | 9.5(5.9-13.1) | 24.5(20.4-28.6) | 61.3(51.6-71.0) | 49.5(40.3-58.7) | 24.8(15.1-34.5) |
| Sex by age (years) |  |  |  |  |  |  |  |  |  |
| Men |  |  |  |  |  |  |  |  |  |
| 20-39 (n=732) | 0.4(0.0-1.1)^†^ | 1.9(0.2-3.6)^†^ | 3.0(0.0-5.9)^†^ | 5.2(1.1-9.3)^†^ | 0.5(0.0-1.5)^†^ | 5.8(1.1-10.4)^†^ | 91.0(77.5-100.0)^‡^ | 36.0(10.6-61.3)^†^ | 22.8(1.4-44.2)^†^ |
| 40-59 (n=1555) | 1.7(1.2-2.2)^‡^ | 4.3(2.9-5.7)^‡^ | 5.9(3.8-8.0)^‡^ | 12.0(8.9-15.0)^‡^ | 6.9(3.8-10.1)^‡^ | 18.9(14.3-23.5)^‡^ | 63.3(51.8-74.8) | 35.9(28.3-43.5) | 15.3(10.2-20.4) |
| 60-74 (n=554) | 0.9(0.0-1.9)^†^ | 7.5(6.0-9.1) | 6.8(4.0-9.6) | 15.3(12.3-18.2) | 9.0(4.9-13.2) | 24.3(20.6-28.0) | 62.9 (49.3-76.5) | 49.2(34.6-63.9) | 25.7(12.0-39.4) |
| Women |  |  |  |  |  |  |  |  |  |
| 20-39 (n=1057) | 1.0(0.1-2.0)^†^ | 1.4(0.1-2.6)^†^ | 1.6(0.3-2.9)^†^ | 4.0(2.5-5.5) | 2.0(0.4-3.6)^†^ | 6.0(3.9-8.1) | 66.6(46.6-86.6) | 34.0(2.9-65.0)^†^ | 17.5(0.0-35.1)^†^ |
| 40-59 (n=3018) | 1.1(0.8-1.4) | 2.9(2.0-3.7) | 3.5(2.5-4.4) | 7.4(5.8-9.0) | 4.9(2.9-6.9) | 12.3(10.5-14.1) | 60.3(47.2-73.3) | 38.5(31.3-45.7) | 11.9(7.6-16.3) |
| 60-74 (n=997) | 1.7(0.8-2.5) | 7.3(5.7-9.0) | 5.8(4.8-6.8) | 14.8(12.3-17.3) | 10.0(5.7-14.2) | 24.7(18.9-30.6) | 59.7(50.6-68.8) | 49.7(44.3-55.2) | 23.9(14.7-33.1) |

FPG, fasting plasma glucose; n, number of participants. *Total diabetes includes diagnosed diabetes (determined by self-report on interview) and undiagnosed diabetes (isolated fasting diabetes, isolated 2h post-load diabetes, and combined fasting and post-load diabetes). ^†^Relative SE >30%: the CI is wide, relative to the size of estimate. ^‡^*P* <0.05 when compared to the prevalence or proportion in women.
