# Supplementary material for: Optimal Cut-Off Points of Fasting Plasma Glucose for Two-Step Strategy in Estimating Prevalence and Screening Undiagnosed Diabetes and Pre-Diabetes in Harbin, China
Source: PLoS One. 2015 Mar 18;10(3):e0119510. doi: 10.1371/journal.pone.0119510 (PMC4364753; doi:10.1371/journal.pone.0119510)
Supplement: S2 Table — (DOC) [file pone.0119510.s002.doc]

**S2 Table. Prevalence of pre-diabetes, proportion of isolated IGT in total pre-diabetes, and missed diagnosis of pre-diabetes using optimal FPG cut-off points for two-step strategy by age and sex based on ADA and WHO criteria, in urban Harbin of China, 2008 (%, 95% CI)**

|  | Isolated IFG | Isolated IGT | Combined IFG and IGT | Total pre-diabetes^*^ | Total diabetes and pre-diabetes | Proportion of isolated IGT in total pre-diabetes | Proportion of isolated IGT which FPG <5.3 mmol/l (ADA criteria) or <5.0 mmol/l (WHO criteria ) in total pre-diabetes |
| --- | --- | --- | --- | --- | --- | --- | --- |
| ADA criteria^†^ |  |  |  |  |  |  |  |
| Number | 358 | 479 | 195 | 1032 | 2177 |  |  |
| 20-74 | 4.2(2.7-5.7) | 5.7(4.5-6.8) | 2.1(1.4-2.9) | 12.0(9.4-14.7) | 24.8(19.8-29.8) | 47.2(38.9-55.6) | 37.3(29.3-45.3) |
| Sex |  |  |  |  |  |  |  |
| Men | 4.8(2.7-7.0) | 5.9(4.3-7.4) | 2.2(1.2-3.2) | 12.9(8.8-16.9) | 26.7(17.6-35.8) | 45.6(38.5-52.6) | 35.1(27.4-42.9) |
| Women | 3.6(2.4-4.7) | 5.5(4.2-6.8) | 2.1(1.4-2.8) | 11.2(9.4-13.0) | 22.8(20.4-25.3) | 49.2(37.7-60.7) | 39.8(30.1-49.5) |
| Age-specific groups (years) |  |  |  |  |  |  |  |
| 20-39 | 2.7(1.2-4.1) | 3.5(2.1-5.0) | 0.9(0.5-1.3) | 7.1(5.2-8.9) | 13.0(9.2-16.7) | 49.6(33.9-65.2) | 38.2(22.7-53.7) |
| 40-59 | 5.6(3.5-7.8) | 6.2(5.2-7.1) | 3.2(2.2-4.3) | 15.0(12.4-17.6) | 30.6(26.0-35.1) | 41.0(32.5-49.5) | 33.2(24.7-41.7) |
| 60-74 | 4.6(3.3-5.9) | 10.5(9.0-12.0) | 2.8(1.9-3.8) | 17.9(15.9-19.9) | 42.4(39.0-45.9) | 58.4(51.2-65.7) | 45.2(38.5-52.0) |
| Sex by age (years) |  |  |  |  |  |  |  |
| Men |  |  |  |  |  |  |  |
| 20-39 | 3.5 (0.9-6.2)^§^ | 3.3 (2.3-4.4) | 0.9 (0.3-1.5)^§^ | 7.8(4.4-11.1) | 13.5(6.5-20.6) | 42.9(30.7-55.0) | 27.8(16.1-39.5)|| |
| 40-59 | 6.5 (4.0-9.0)|| | 6.9 (5.5-8.3)|| | 3.8 (2.4-5.1)|| | 17.2(13.6-20.8)|| | 36.1(28.6-43.6)|| | 40.4(33.4-47.4) | 31.9(23.7-40.1) |
| 60-74 | 4.2 (2.6-5.9) | 10.5 (8.0-12.9) | 1.8 (0.6-3.0) | 16.6(13.4-19.7) | 40.8(37.1-44.5) | 63.3(52.9-73.7)|| | 53.9(43.2-64.5)|| |
| Women |  |  |  |  |  |  |  |
| 20-39 | 1.7 (0.5-3.0)^§^ | 3.7(1.3-6.1) | 0.9 (0.1-1.6)^§^ | 6.3(4.1-8.5) | 12.3(9.4-15.3) | 58.7(33.0-84.5) | 52.4(31.5-73.2) |
| 40-59 | 4.8 (2.9-6.7) | 5.4 (4.3-6.5) | 2.7 (1.8-3.6) | 12.9(10.7-15.2) | 25.2(22.0-28.5) | 41.8(30.6-53.0) | 34.9(24.7-45.0) |
| 60-74 | 5.0 (3.4-6.6) | 10.5 (8.5-12.4) | 3.8 (2.6-5.1) | 19.3(16.2-22.4) | 44.0(38.7-49.4) | 54.2(49.0-59.5) | 37.9(31.4-44.4) |
| WHO criteria^‡^ |  |  |  |  |  |  |  |
| Number | 108 | 578 | 96 | 782 | 1927 |  |  |
| 20-74 | 1.2(0.7-1.7) | 6.8(5.6-8.1) | 1.0(0.4-1.6) | 9.0(7.2-10.9) | 21.8(17.5-26.1) | 75.4(68.0-82.7) | 38.1(32.4-43.7) |
| Sex |  |  |  |  |  |  |  |
| Men | 1.3(0.7-1.9) | 7.1(5.4-8.8) | 0.9(0.2-1.7)^§^ | 9.3(6.7-11.9) | 23.1(15.3-31.0) | 76.2(69.8-82.5) | 36.5(30.9-42.0) |
| Women | 1.1(0.4-1.8) | 6.5(5.2-7.8) | 1.1(0.7-1.5) | 8.8(7.2-10.3) | 20.4(17.9-22.9) | 74.5(64.5-84.5) | 39.8(31.5-48.2) |
| Age-specific groups (years) |  |  |  |  |  |  |  |
| 20-39 | 0.8(0.2-1.4)^§^ | 4.1(2.7-5.4) | 0.3(0.0-0.7)^§^ | 5.2(3.9-6.5) | 11.1(7.8-14.3) | 78.2(62.3-94.1) | 42.6(27.6-57.6) |
| 40-59 | 1.5(0.9-2.2) | 7.9(6.6-9.1) | 1.5(0.9-2.1) | 10.9(9.2-12.6) | 26.4(22.2-30.7) | 72.1(65.7-78.5) | 33.0(25.0-41.0) |
| 60-74 | 1.5(0.8-2.3) | 11.7(9.9-13.5) | 1.6(0.4-2.7)^§^ | 14.8(13.1-16.6) | 39.4(35.3-43.4) | 79.0(69.7-88.3) | 43.4(37.4-49.4) |
| Sex by age (years) |  |  |  |  |  |  |  |
| Men |  |  |  |  |  |  |  |
| 20-39 | 0.8(0.1-1.4)^§^ | 3.9(2.9-4.9) | 0.3(0.0-1.0)^§^ | 5.0(3.6-6.4) | 10.8(5.2-16.3) | 78.3(65.9-90.7) | 32.1(19.1-45.1)|| |
| 40-59 | 1.7(0.9-2.4) | 9.2(7.3-11.1)|| | 1.5(0.7-2.3) | 12.4(9.8-14.9)|| | 31.2(24.3-38.2)|| | 74.2(68.1-80.2) | 31.2(22.9-39.5) |
| 60-74 | 1.9(0.6-3.2)^§^ | 11.2(8.4-13.9) | 1.2(0.0-2.5)^§^ | 14.2(12.0-16.4) | 38.5(34.6-42.4) | 78.5(64.7-92.2) | 52.8(41.3-64.3)|| |
| Women |  |  |  |  |  |  |  |
| 20-39 | 0.8(0.0-2.2)^§^ | 4.2(1.8-6.6) | 0.4(0.0-0.8)^§^ | 5.4(3.2-7.6) | 11.4(8.2-14.7) | 78.1(51.6-100.0) | 53.4(33.4-73.4) |
| 40-59 | 1.4(0.8-2.0) | 6.6(5.4-7.8) | 1.5(1.0-2.0) | 9.5(8.0-11.1) | 21.8(18.7-25.0) | 69.5(60.8-78.2) | 35.3(25.5-45.1) |
| 60-74 | 1.1(0.5-1.8) | 12.3(10.0-14.6) | 2.0(0.6-3.4)^§^ | 15.5(13.1-17.8) | 40.2(34.7-45.7) | 79.5(71.6-87.4) | 34.8(26.1-43.5) |

FPG, fasting plasma glucose; ADA, American Diabetes Association; WHO, World Health Organization; IFG, impaired fasting glucose; IGT, impaired glucose tolerance. ^*^Isolated IFG, isolated IGT, and combined IFG and IGT. Pre-diabetes includes IFG and/or IGT; ^†^IFG using ADA criteria, FPG 5.6 to <7.0 mmol/l; ^‡^IFG using WHO criteria, FPG 6.1 to <7.0 mmol/l; IGT, 2-h PG 7.8 to <11.1 mmol/l. ^§^Relative SE >30%: the CI is wide, relative to the size of estimate. ^||^*P* <0.05 when compared to the prevalence or proportion in women.
