# Supplementary material for: Optimal Cut-Off Points of Fasting Plasma Glucose for Two-Step Strategy in Estimating Prevalence and Screening Undiagnosed Diabetes and Pre-Diabetes in Harbin, China
Source: PLoS One. 2015 Mar 18;10(3):e0119510. doi: 10.1371/journal.pone.0119510 (PMC4364753; doi:10.1371/journal.pone.0119510)
Supplement: S3 Table — (DOC) [file pone.0119510.s003.doc]

**S3 Table. AUCs and screening potential for 2-h PG alone in screening undiagnosed diabetes and/or pre-diabetes with optimal cut-off points**

| Optimal cut-off point (mmol/l) | Positive/Negative^*^ | Sensitivity (%) | Specificity (%) | Likelihood ratio (%) | | Predictive value (%) | | Kappa | AUC (95% CI) | Post-test probability (%) | | | |
| --- | --- | --- | --- | --- | --- | --- | --- | --- | --- | --- | --- | --- | --- |
|  |  |  |  | Positive | Negative | Positive | Negative |  |  | Men | Age >40 years | Obesity | Abdominal obesity |
| >=10.3 for undiagnosed diabetes | 696/6768 | 88.2 | 98.3 | 52.4 | 0.1 | 84.3 | 98.8 | 0.86 | 0.96(0.95-0.97)^§^ | 84.5 | 86.7 | 87.3 | 87.7 |
| >=7.9 for diabetes and pre-diabetes^†^ (ADA criteria) | 1728/5736 | 76.1 | 100.0 | - | 0.2 | 100.0 | 93.3 | 0.81 | 0.92(0.91-0.93)^§^ | 100.0 | 100.0 | 100.0 | 100.0 |
| >=7.9 for diabetes and pre-diabetes^‡^ (WHO criteria) | 1478/5986 | 89.0 | 100.0 | - | 0.1 | 100.0 | 97.3 | 0.91 | 0.96(0.96-0.97)^§^ | 100.0 | 100.0 | 100.0 | 100.0 |
| >=7.1 for pre-diabetes^†^ (ADA criteria) | 1032/5736 | 72.7 | 93.4 | 11.0 | 0.3 | 66.5 | 95.0 | 0.65 | 0.89(0.88-0.90)^§^ | 62.0 | 67.4 | 68.3 | 67.5 |
| >=7.8 for pre-diabetes^‡^ (WHO criteria) | 782/5986 | 87.2 | 99.3 | 124.3 | 0.1 | 94.2 | 98.3 | 0.92 | 0.96(0.95-0.97)^§^ | 92.7 | 94.4 | 94.8 | 94.3 |

AUCs, the area under the receiver-operating characteristic curves; 2-h PG, 2 hour post-load plasma glucose. ^*^Number of participants based on golden standard. Pre-diabetes includes IFG and/or IGT. ^†^IFG using ADA criteria, FPG 5.6 to <7.0 mmol/l; ^‡^IFG using WHO criteria, FPG 6.1 to <7.0 mmol/l; IGT, 2-h PG 7.8 to <11.1 mmol/l. ^§^*P* <0.001 compared with area under curve of fasting plasma glucose.
