# Supplementary material for: Optimal Cut-Off Points of Fasting Plasma Glucose for Two-Step Strategy in Estimating Prevalence and Screening Undiagnosed Diabetes and Pre-Diabetes in Harbin, China
Source: PLoS One. 2015 Mar 18;10(3):e0119510. doi: 10.1371/journal.pone.0119510 (PMC4364753; doi:10.1371/journal.pone.0119510)
Supplement: S4 Table — (DOC) [file pone.0119510.s004.doc]

**S4 Table. The cost-effectiveness of two-step strategy at different fasting plasma glucose cut-off points in screening IGT, undiagnosed diabetes and/or pre-diabetes.**

| FPG (mmol/l) | Sensitivity (%) | Specificity (%) | Number | | |  | Cost per case identified ( ¥/case ) | | |
| --- | --- | --- | --- | --- | --- | --- | --- | --- | --- |
|  |  |  | participants | participants that need perform further OGTT test | Cases identified |  | Medical | Non-medical | Total |
| Screening undiagnosed diabetes alone^&^ |  |  |  |  |  |  |  |  |  |
| ≥4.6 | 95.3 | 53.1 | 3742 | 3328 | 660 |  | 148 | 233 | 380 |
| ≥4.9 | 91.8 | 70.2 | 2590 | 2176 | 638 |  | 132 | 191 | 322 |
| ≥5.0 | 90.4 | 75.0 | 2257 | 1843 | 626 |  | 128 | 180 | 308 |
| ≥5.2 | 88.1 | 82.3 | 1766 | 1352 | 611 |  | 121 | 162 | 284 |
| ≥5.3 | 87.1 | 85.4 | 1550 | 1136 | 604 |  | 119 | 154 | 273 |
| ≥5.5 | 83.5 | 89.7 | 1249 | 835 | 579 |  | 118 | 147 | 264 |
| ≥5.6* | 82.0 | 91.6 | 1121 | 707 | 568 |  | 117 | 143 | 261 |
| ≥5.8 | 78.0 | 94.1 | 928 | 514 | 541 |  | 119 | 141 | 260 |
| ≥6.1 | 73.6 | 96.9 | 715 | 301 | 511 |  | 121 | 137 | 258 |
| ≥6.4 | 70.1 | 98.3 | 597 | 183 | 486 |  | 124 | 138 | 262 |
| ≥6.7 | 65.2 | 99.4 | 487 | 73 | 452 |  | 131 | 142 | 272 |
| ≥7.0 | 59.8 | 100.0 | 414 | 0 | 414 |  | 141 | 150 | 290 |
| Screening both diabetes and pre-diabetes^†&^  (ADA criteria) |  |  |  |  |  |  |  |  |  |
| ≥4.6 | 88.7 | 59.8 | 3742 | 3328 | 1522 |  | 64 | 101 | 165 |
| ≥4.9 | 81.9 | 78.4 | 2590 | 2176 | 1406 |  | 60 | 87 | 146 |
| ≥5.0 | 79.2 | 83.4 | 2257 | 1843 | 1359 |  | 59 | 83 | 142 |
| ≥5.2 | 74.7 | 90.9 | 1766 | 1352 | 1284 |  | 58 | 77 | 135 |
| ≥5.3* | 72.4 | 94.4 | 1550 | 1136 | 1243 |  | 58 | 75 | 133 |
| ≥5.5 | 67.4 | 98.1 | 1249 | 835 | 1157 |  | 59 | 73 | 132 |
| ≥5.6 | 65.3 | 99.8 | 1121 | 707 | 1121 |  | 59 | 73 | 132 |
| ≥5.8 | 54.6 | 100.0 | 928 | 514 | 928 |  | 69 | 82 | 151 |
| ≥6.1 | 41.9 | 100.0 | 715 | 301 | 715 |  | 86 | 98 | 185 |
| ≥6.4 | 34.9 | 100.0 | 597 | 183 | 597 |  | 101 | 112 | 213 |
| ≥6.7 | 28.5 | 100.0 | 487 | 73 | 487 |  | 121 | 131 | 253 |
| ≥7.0 | 24.3 | 100.0 | 414 | 0 | 414 |  | 141 | 150 | 290 |
| Screening both diabetes and pre-diabetes^†#^  (ADA criteria) |  |  |  |  |  |  |  |  |  |
| ≥4.6 | 88.7 | 59.8 | 3742 | 2621 | 1522 |  | 59 | 88 | 147 |
| ≥4.9 | 81.9 | 78.4 | 2590 | 1469 | 1406 |  | 54 | 73 | 127 |
| ≥5.0 | 79.2 | 83.4 | 2257 | 1136 | 1359 |  | 53 | 69 | 121 |
| ≥5.2 | 74.7 | 90.9 | 1766 | 645 | 1284 |  | 51 | 62 | 113 |
| ≥5.3* | 72.4 | 94.4 | 1550 | 429 | 1243 |  | 51 | 59 | 110 |
| ≥5.5 | 67.4 | 98.1 | 1249 | 128 | 1157 |  | 52 | 57 | 108 |
| ≥5.6 | 65.3 | 99.8 | 1121 | 0 | 1121 |  | 52 | 55 | 107 |
| ≥5.8 | 54.6 | 100.0 | 928 | 0 | 928 |  | 63 | 67 | 130 |
| ≥6.1 | 41.9 | 100.0 | 715 | 0 | 715 |  | 81 | 87 | 168 |
| ≥6.4 | 34.9 | 100.0 | 597 | 0 | 597 |  | 98 | 104 | 201 |
| ≥6.7 | 28.5 | 100.0 | 487 | 0 | 487 |  | 120 | 127 | 247 |
| ≥7.0 | 24.3 | 100.0 | 414 | 0 | 414 |  | 141 | 150 | 290 |
| Screening both diabetes and pre-diabetes^‡&^  (WHO criteria) |  |  |  |  |  |  |  |  |  |
| ≥4.6 | 86.7 | 57.3 | 3742 | 3328 | 1272 |  | 77 | 121 | 197 |
| ≥4.9 | 78.9 | 75.1 | 2590 | 2176 | 1156 |  | 73 | 105 | 178 |
| ≥5.0 | 75.6 | 79.9 | 2257 | 1843 | 1109 |  | 72 | 102 | 174 |
| ≥5.2 | 70.4 | 87.1 | 1766 | 1352 | 1034 |  | 72 | 96 | 168 |
| ≥5.3* | 68.0 | 90.2 | 1550 | 1136 | 993 |  | 72 | 94 | 166 |
| ≥5.5 | 61.9 | 94.0 | 1249 | 835 | 907 |  | 75 | 94 | 169 |
| ≥5.6 | 59.4 | 95.6 | 1121 | 707 | 871 |  | 76 | 93 | 170 |
| ≥5.8 | 54.7 | 97.8 | 928 | 514 | 802 |  | 80 | 95 | 175 |
| ≥6.1 | 48.7 | 99.9 | 715 | 301 | 715 |  | 86 | 98 | 185 |
| ≥6.4 | 40.8 | 100.0 | 597 | 183 | 597 |  | 101 | 112 | 213 |
| ≥6.7 | 33.3 | 100.0 | 487 | 73 | 487 |  | 121 | 131 | 253 |
| ≥7.0 | 28.4 | 100.0 | 414 | 0 | 414 |  | 141 | 150 | 290 |
| Screening both diabetes and pre-diabetes^‡#^  (WHO criteria) |  |  |  |  |  |  |  |  |  |
| ≥4.6 | 86.7 | 57.3 | 3742 | 3027 | 1272 |  | 74 | 114 | 188 |
| ≥4.9 | 78.9 | 75.1 | 2590 | 1875 | 1156 |  | 70 | 98 | 168 |
| ≥5.0 | 75.6 | 79.9 | 2257 | 1542 | 1109 |  | 69 | 94 | 163 |
| ≥5.2 | 70.4 | 87.1 | 1766 | 1051 | 1034 |  | 68 | 88 | 156 |
| ≥5.3* | 68.0 | 90.2 | 1550 | 835 | 993 |  | 69 | 86 | 154 |
| ≥5.5 | 61.9 | 94.0 | 1249 | 534 | 907 |  | 71 | 85 | 156 |
| ≥5.6 | 59.4 | 95.6 | 1121 | 406 | 871 |  | 72 | 84 | 156 |
| ≥5.8 | 54.7 | 97.8 | 928 | 213 | 802 |  | 76 | 85 | 160 |
| ≥6.1 | 48.7 | 99.9 | 715 | 0 | 715 |  | 81 | 87 | 168 |
| ≥6.4 | 40.8 | 100.0 | 597 | 0 | 597 |  | 98 | 104 | 201 |
| ≥6.7 | 33.3 | 100.0 | 487 | 0 | 487 |  | 120 | 127 | 247 |
| ≥7.0 | 28.4 | 100.0 | 414 | 0 | 414 |  | 141 | 150 | 290 |
| Screening pre-diabetes^† &^alone  (ADA criteria) |  |  |  |  |  |  |  |  |  |
| ≥4.6 | 86.6 | 53.6 | 3742 | 3328 | 862 |  | 113 | 178 | 291 |
| ≥4.9 | 77.3 | 73.9 | 2590 | 2176 | 768 |  | 109 | 159 | 268 |
| ≥5.0 | 74.4 | 79.4 | 2257 | 1843 | 733 |  | 109 | 154 | 263 |
| ≥5.2 | 67.8 | 88.0 | 1766 | 1352 | 673 |  | 110 | 147 | 258 |
| ≥5.3* | 62.9 | 94.1 | 1550 | 1136 | 639 |  | 112 | 146 | 258 |
| ≥5.5 | 56.6 | 98.1 | 1249 | 835 | 578 |  | 118 | 147 | 265 |
| ≥5.6 | 54.0 | 99.8 | 1121 | 707 | 553 |  | 120 | 147 | 268 |
| ≥5.8 | 38.8 | 100.0 | 928 | 514 | 387 |  | 166 | 197 | 363 |
| ≥6.1 | 20.5 | 100.0 | 715 | 301 | 204 |  | 303 | 344 | 647 |
| ≥6.4 | 11.1 | 100.0 | 597 | 183 | 111 |  | 544 | 604 | 1147 |
| ≥6.7 | 3.7 | 100.0 | 487 | 73 | 35 |  | 1688 | 1827 | 3515 |
| ≥7.0 | 0.3 | 100.0 | 414 | 0 | 0 |  | - | - | - |
| Screening pre-diabetes^‡^ ^&^alone  (WHO criteria) |  |  |  |  |  |  |  |  |  |
| ≥4.6 | 82.4 | 51.3 | 3742 | 3328 | 612 |  | 159 | 251 | 410 |
| ≥4.9 | 70.1 | 70.8 | 2590 | 2176 | 518 |  | 162 | 235 | 397 |
| ≥5.0* | 66.8 | 75.8 | 2257 | 1843 | 483 |  | 166 | 233 | 399 |
| ≥5.2 | 54.7 | 87.1 | 1766 | 1352 | 423 |  | 175 | 234 | 410 |
| ≥5.3 | 51.0 | 90.2 | 1550 | 1136 | 389 |  | 184 | 240 | 424 |
| ≥5.5 | 42.7 | 94.0 | 1249 | 835 | 328 |  | 208 | 259 | 466 |
| ≥5.6 | 39.3 | 95.6 | 1121 | 707 | 303 |  | 220 | 269 | 488 |
| ≥5.8 | 34.0 | 97.8 | 928 | 514 | 261 |  | 246 | 292 | 538 |
| ≥6.1 | 26.5 | 99.9 | 715 | 301 | 204 |  | 303 | 344 | 647 |
| ≥6.4 | 14.7 | 100.0 | 597 | 183 | 111 |  | 544 | 604 | 1147 |
| ≥6.7 | 4.9 | 100.0 | 487 | 73 | 35 |  | 1688 | 1827 | 3515 |
| ≥7.0 | 0.4 | 100.0 | 414 | 0 | 0 |  | - | - | - |
| Screening IGT alone^&^ |  |  |  |  |  |  |  |  |  |
| ≥4.6 | 75.8 | 56.3 | 3742 | 3328 | 504 |  | 193 | 305 | 498 |
| ≥4.9* | 62.2 | 73.8 | 2590 | 2176 | 410 |  | 205 | 297 | 502 |
| ≥5.0 | 56.5 | 78.5 | 2257 | 1843 | 375 |  | 213 | 300 | 514 |
| ≥5.2 | 47.5 | 85.5 | 1766 | 1352 | 315 |  | 236 | 315 | 550 |
| ≥5.3 | 43.2 | 88.6 | 1550 | 1136 | 281 |  | 255 | 332 | 587 |
| ≥5.5 | 33.5 | 92.3 | 1249 | 835 | 220 |  | 309 | 386 | 695 |
| ≥5.6 | 29.5 | 93.9 | 1121 | 707 | 195 |  | 341 | 417 | 759 |
| ≥5.8 | 23.4 | 96.0 | 928 | 514 | 153 |  | 420 | 497 | 918 |
| ≥6.1 | 14.7 | 98.2 | 715 | 301 | 96 |  | 643 | 732 | 1375 |
| ≥6.4 | 9.1 | 99.1 | 597 | 183 | 58 |  | 1041 | 1155 | 2196 |
| ≥6.7 | 3.1 | 99.7 | 487 | 73 | 20 |  | 2954 | 3198 | 6152 |
| ≥7.0 | 0.2 | 100.0 | 414 | 0 | 0 |  | - | - | - |

FPG, fasting plasma glucose; ADA, American Diabetes Association; WHO, World Health Organization; IGT, impaired glucose tolerance. ^†^IFG using ADA criteria, FPG 5.6 to <7.0 mmol/l; ^‡^IFG using WHO criteria, FPG 6.1 to <7.0 mmol/l; IGT, 2-h PG 7.8 to <11.1 mmol/l. *Optimal cut-off points of fasting plasma glucose for two-step strategy in estimating prevalence and screening undiagnosed diabetes and pre-diabetes in our study. ^&^Further OGTT was not conducted for subjects with FPG ≥7.0 mmol/l in the case of screening for undiagnosed diabetes or both diabetes and pre-diabetes or IGT or pre-diabetes alone. ^#^Further OGTT was not conducted for subjects with FPG ≥5.6 mmol/l (≥6.1mmol/l) in the case of screening for both diabetes and pre-diabetes using ADA criteria (WHO criteria).
