# Supplementary material for: Optimal Cut-Off Points of Fasting Plasma Glucose for Two-Step Strategy in Estimating Prevalence and Screening Undiagnosed Diabetes and Pre-Diabetes in Harbin, China
Source: PLoS One. 2015 Mar 18;10(3):e0119510. doi: 10.1371/journal.pone.0119510 (PMC4364753; doi:10.1371/journal.pone.0119510)
Supplement: S5 Table — (DOC) [file pone.0119510.s005.doc]

**S5 Table. Mean FPG and mean 2 hour post-load plasma glucose in OGTT by group of plasma glucose categories and sex based on ADA and WHO criteria. (mmol/l, 95% CI)**

|  | Normoglycemia | Isolated IFG | Isolated IGT | Combined IFG and IGT | IGT | Pre-diabetes^*^ | Undiagnosed diabetes | Diagnosed diabetes |
| --- | --- | --- | --- | --- | --- | --- | --- | --- |
| ADA criteria^†^ |  |  |  |  |  |  |  |  |
| Men |  |  |  |  |  |  |  |  |
| Number | 1967 | 155 | 183 | 73 | 256 | 411 | 293 | 170 |
| Mean FPG | 4.4(4.3-4.5) | 5.9(5.8-6.0) | 4.8(4.7-4.9) | 6.0(5.9-6.2) | 5.1(5.0-5.2) | 5.4(5.3-5.5) | 8.2(8.0-8.5) | 7.6(7.3-7.9) |
| Mean 2-h PG | 4.9(4.6-5.1) | 5.7(5.4-6.1) | 9.0(8.8-9.1) | 9.4(9.2-9.5) | 9.1(8.9-9.2) | 7.8(7.5-8.2) | 15.3(14.5-16.0) | 12.1(9.7-14.5) |
| Women |  |  |  |  |  |  |  |  |
| Number | 3769 | 203 | 296 | 122 | 418 | 621 | 403 | 279 |
| Mean FPG | 4.4(4.3-4.5) | 6.0(5.9-6.1) | 4.7(4.6-4.8) | 6.2(6.1-6.2) | 5.1(4.9-5.3) | 5.4(5.2-5.6) | 8. 0(7.5-8.5) | 7.8(7.1-8.6) |
| Mean 2-h PG | 5.1(5.0-5.2) | 6.0(5.8-6.2) | 9.0(8.9-9.2) | 9.2(9.0-9.5) | 9.1(9.0-9.2) | 8.1(7.8-8.4) | 14.7(13.8-15.6) | 12.6(10.1-15.0) |
| Total |  |  |  |  |  |  |  |  |
| Number | 5736 | 358 | 479 | 195 | 674 | 1032 | 696 | 449 |
| Mean FPG | 4.4(4.3-4.5) | 5.9(5.9-6.0) | 4.7(4.7-4.8) | 6.1(6.0-6.2) | 5.1(5.0-5.3) | 5.4(5.3-5.5) | 8.1(7.9-8.4) | 7.7(7.3-8.2) |
| Mean 2-h PG | 5.0(4.8-5.2) | 5.8(5.6-6.0) | 9.0(8.9-9.1) | 9.3(9.1-9.5) | 9.1(9.0-9.2) | 7.9(7.7-8.2) | 15.0(14.4-15.7) | 12.3(10.0-14.7) |
| WHO criteria^‡^ |  |  |  |  |  |  |  |  |
| Men |  |  |  |  |  |  |  |  |
| Number | 2077 | 45 | 226 | 30 | 256 | 301 | 293 | 170 |
| Mean FPG | 4.4(4.3-4.6) | 6.4(6.3-6.4) | 5.0(4.9-5.0) | 6.4(6.3-6.5) | 5.1(5.0-5.2) | 5.3(5.2-5.4) | 8.2(8.0-8.5) | 7.6(7.3-7.9) |
| Mean 2-h PG | 4.9(4.6-5.2) | 5.6(5.2-6.1) | 9.0(8.9-9.2) | 9.6(9.4-9.8) | 9.1(8.9-9.2) | 8.6(8.4-8.8) | 15.3(14.5-16.0) | 12.1(9.7-14.5) |
| Women |  |  |  |  |  |  |  |  |
| Number | 3909 | 63 | 352 | 66 | 418 | 481 | 403 | 279 |
| Mean FPG | 4.4(4.4-4.5) | 6.4(6.4-6.5) | 4.9(4.7-5.0) | 6.5(6.4-6.5) | 5.1(4.9-5.3) | 5.3(5.0-5.5) | 8.0(7.5-8.5) | 7.8(7.1-8.6) |
| Mean 2-h PG | 5.1(5.1-5.2) | 6.0(5.7-6.3) | 9.0(8.9-9.2) | 9.3(9.0-9.6) | 9.1(9.0-9.2) | 8.7(8.4-8.9) | 14.7(13.9-15.5) | 12.6(10.1-15.0) |
| Total |  |  |  |  |  |  |  |  |
| Number | 5986 | 108 | 578 | 96 | 674 | 782 | 696 | 449 |
| Mean FPG | 4.4(4.3-4.6) | 6.4(6.4-6.4) | 4.9(4.8-5.0) | 6.4(6.4-6.5) | 5.1(5.0-5.3) | 5.3(5.1-5.4) | 8.1(7.9-8.4) | 7.7(7.3-8.2) |
| Mean 2-h PG | 5.0(4.8-5.2) | 5.8(5.5-6.1) | 9.0(8.9-9.1) | 9.4(9.2-9.6) | 9.1(9.0-9.2) | 8.6(8.5-8.8) | 15.0(14.4-15.7) | 12.3(10.0-14.7) |

FPG, fasting plasma glucose; 2-h PG, 2 hour post-load plasma glucose; ADA, American Diabetes Association; WHO, World Health Organization; IFG, impaired fasting glucose; IGT, impaired glucose tolerance. ^*^Isolated IFG, isolated IGT, and combined IFG and IGT; ^†^IFG using ADA criteria, FPG 5.6 to <7.0 mmol/l; ^‡^IFG using WHO criteria, FPG 6.1 to <7.0 mmol/l; IGT, 2-h PG 7.8 to <11.1 mmol/l.
